# Increased Inflammation and Unchanged Density of Synaptic Vesicle Glycoprotein 2A (SV2A) in the Postmortem Frontal Cortex of Alzheimer’s Disease Patients

**DOI:** 10.3389/fncel.2019.00538

**Published:** 2019-12-05

**Authors:** Athanasios Metaxas, Camilla Thygesen, Sanne R. R. Briting, Anne M. Landau, Sultan Darvesh, Bente Finsen

**Affiliations:** ^1^Department of Neurobiology Research, Institute of Molecular Medicine, University of Southern Denmark, Odense, Denmark; ^2^Translational Neuropsychiatry Unit, Aarhus University, Aarhus, Denmark; ^3^Department of Nuclear Medicine and PET-Center, Aarhus University, Aarhus, Denmark; ^4^Department of Medical Neuroscience, Dalhousie University, Halifax, NS, Canada; ^5^Department of Medicine, Neurology, and Geriatric Medicine, Dalhousie University, Halifax, NS, Canada

**Keywords:** neuroinflammation, translocator protein, [^3^H]PK11195, synapses, synaptic vesicle glycoprotein 2A, [^3^H]UCB-J, amyloid, tau

## Abstract

Sections from the middle frontal gyrus (Brodmann area 46) of autopsy-confirmed Alzheimer’s disease (AD) patients and non-demented subjects were examined for the prevalence of hallmark AD pathology, including amyloid-β (Aβ) plaques, phosphorylated tau (pTau) tangles, neuroinflammation and synaptic loss (*n* = 7 subjects/group). Dense-core deposits of Aβ were present in all AD patients (7/7) and some non-demented subjects (3/7), as evidenced by 6E10 immunohistochemistry. Levels of Aβ immunoreactivity were higher in AD vs. non-AD cases. For pTau, AT8-positive neurofibrillary tangles and threads were exclusively observed in AD patient tissue. Levels of [^3^H]PK11195 binding to the translocator protein (TSPO), a marker of inflammatory processes, were elevated in the gray matter of AD patients compared to non-demented subjects. Levels of [^3^H]UCB-J binding to synaptic vesicle glycoprotein 2A (SV2A), a marker of synaptic density, were not different between groups. In AD patients, pTau immunoreactivity was positively correlated with [^3^H]PK11195, and negatively correlated with [^3^H]UCB-J binding levels. No correlation was observed between Aβ immunoreactivity and markers of neuroinflammation or synaptic density. These data demonstrate a close interplay between tau pathology, inflammation and SV2A density in AD, and provide useful information on the ability of neuroimaging biomarkers to diagnose AD dementia.

## Introduction

Despite considerable advances in biological fluid and brain imaging biomarkers, autopsy remains the most reliable means of obtaining a definitive diagnosis of dementia due to Alzheimer’s disease (AD). The diagnosis is based on the microscopic identification of hallmark AD pathology in the brain, most notably the deposition of amyloid-beta (Aβ) peptides into plaques, the accumulation of hyperphosphorylated tau (pTau) protein into neurofibrillary tangles and neurodegeneration (Jack et al., 2018). In addition, a brain-specific form of low-grade, chronic inflammation is known to accompany the progression of AD (Heneka et al., 2015).

It is now recognized that symptomatic AD is preceded by a long preclinical phase, which is characterized by the insidious accumulation of AD neuropathologic lesions. Biomarker studies in subjects with normal cognition indicate that the accumulation of Aβ may precede the onset of memory decline by at least a decade (Jansen et al., 2018). Moreover, autopsy studies demonstrate that aggregated Aβ and pTau can be detected in certain brain areas of cognitively intact individuals by the third to fourth decades of life (Braak and Braak, 1997; Braak et al., 2011). As the pathognomonic lesions of AD are present in a significant proportion of individuals with normal cognition, dissociating AD from physiological brain aging represents a major challenge in the dementia research field. Of note, the assumption that Aβ and pTau biomarker-positive subjects are on a path to developing dementia remains a point of contention (Nelson et al., 2011; Franceschi et al., 2018).

In the present study, markers of neuroinflammation and synaptic density were evaluated for their ability to distinguish between autopsy-confirmed AD patients and non-AD subjects. Levels of the translocator protein (TSPO) and synaptic vesicle glycoprotein 2A (SV2A) were measured by autoradiography in sections from the middle frontal gyrus (Brodmann area 46) of AD patients and non-demented subjects. The middle frontal gyrus was chosen for examination based on its susceptibility to both age- and AD-related atrophy (Bakkour et al., 2013), and because of its enhanced vulnerability to Aβ deposition in both cognitively impaired and healthy individuals (Rodrigue et al., 2009). Our results show that there is increased inflammation in Brodmann area 46 in AD, while SV2A levels remain unchanged. These data provide useful insights into the molecular neuropathology of AD and can inform the debate over the ability of imaging biomarkers to confirm a clinical AD diagnosis.

## Materials and Methods

### Ethics Statement

The study was carried out in accordance with the recommendations of the Danish Biomedical Research Ethical Committee for the Region of Southern Denmark (Project Id. S-20160036) and the Nova Scotia Health Authority Research Ethics Board in Halifax, NS, Canada. Written, informed consent forms were obtained for all subjects, in accordance with the Declaration of Helsinki. Samples were transported to the University of Southern Denmark from the Maritime Brain Tissue Bank, Department of Medical Neuroscience, Faculty of Medicine, Dalhousie University, Halifax, Canada.

### Subjects and Tissue Sectioning

Snap-frozen samples from the middle frontal gyrus of autopsy-confirmed AD patients and non-demented subjects were used (*n* = 7/group; Table 1). The groups were matched for sex (3 females, 4 males) and age (median: AD = 79 years, range: 64–92; non-AD = 73 years, range: 47–86; *U* = 19.5, *P* = 0.56). Brain weight at time of removal was lower for AD, compared to non-demented subjects (median: AD = 1151 g, range: 950–1293; non-AD = 1300 g, range: 1210–1451; *U* = 5.0, *P* = 0.01). Histopathological examination of the brain was performed for all subjects.

**TABLE 1:** Subject characteristics.

**No**	**Age range (years)**	**Brain weight (g)**	**PMI (h)**	**CERAD**	**Braak**	**Cause of death**	**Co-morbidities**	**Study group**
1	61–65	1100	24	Frequent (C3)	VI	N/A	N/A	AD
2	76–80	1250	9.5	Moderate (C2)	VI	Pneumonia, Dehydration	Type-2 diabetes, HTN	AD
3	81–85	950	9	Frequent (C3)	V	Inanition	None	AD
4	91–95	1149	64	Frequent (C3)	VI	N/A	None	AD
5	76–80	1200	9	Frequent (C3)	IV	N/A	Emphysema, hyperthyroidism	AD
6	71–75	1151	6.5	Frequent (C3)	VI	Sepsis	Cardiovascular (atherosclerosis)	AD
7	81–85	1293	17.5	Moderate-Frequent (C2-3)	V	N/A	Giant cell arteritis	AD
8	46–50	1275	N/A	None (C0)	0	N/A	None	Non-AD
9	81–85	1210	5.5	Sparse (C1)	0	Surgery complications	Type-2 diabetes, HTN, cardiovascular (atrial fibrillation)	Non-AD
10	81–85	1235	N/A	Moderate (C2)	II	Cancer (breast)	None	Non-AD
11	71–75	1350	36	Sparse (C1)	0	Pancreatitis	None	Non-AD
12	86–90	1300	24	Sparse (C1)	0	Cancer (abdominal)	None	Non-AD
13	46–50	1410	N/A	None (C0)	0	Myocardial infarction	Type-1 diabetes, HTN	Non-AD
14	71–75	1451	68.5	Sparse-moderate (C1-2)	I–II	Pulmonary embolism	None	Non-AD

*PMI, post-mortem interval; N/A, information not available; HTN, hypertension.*

Consecutive, 20 μm-thick sections were collected at −20°C using a Leica CM3050S cryostat (Leica Biosystems). The sections were mounted onto Superfrost^TM^ Plus slides and kept at −80°C until use.

### Aβ and pTau Immunohistochemistry

Frozen sections were fixed in 4% paraformaldehyde overnight and processed for Aβ and pTau immunohistochemistry using standard protocols (Metaxas et al., 2018). Biotinylated mouse primary antibodies against human Aβ (clone 6E10, 2 μg/mL; 803008, BioLegend^®^) and pTau (clone AT8, 0.2 μg/mL; MN1020B, Thermo Fisher Scientific) were diluted in Tris–buffered saline (TBS; pH 7.4), containing 10% fetal bovine serum. Sections were incubated with primary antibodies at −4°C overnight, followed by washing and incubation for 2 h at room temperature with HRP-Streptavidin (1:200; RPN1231V, GE Healthcare). The slides were developed in TBS (pH 7.4), containing 3,3’-diaminobenzidine (DAB; 0.05%) and H_2_O_2_ (0.01%). Biotin-labeled mouse IgG1 (MG115, Thermo Fisher Scientific), diluted to the same concentration as the primary antibodies, was used for isotype control. The sections were dehydrated in ascending concentration of ethanol, cleared in xylene and cover-slipped with PERTEX^®^ (Histolab Products AB). Digital images were obtained under the 4x objective of an Olympus DP80 Dual Monochrome CCD camera, mounted on a motorized BX63 Olympus microscope. For analysis, the images were converted to 8-bit and manually thresholded in ImageJ (version 1.51; National Institutes of Health, MD, United States). The particle analysis plugin was used to measure the percentage of immunoreactive area relative to total image area (% area fraction).

### TSPO and SV2A Autoradiography

Sections were thawed to room temperature and prewashed in 50 mM Tris–HCl buffer (pH 7.4), containing 150 mM NaCl, 5 mM KCl, 1.5 mM MgCl_2_, and 1.5 mM CaCl_2_ (assay buffer; 2 × 10 min). For TSPO, the sections were incubated for 2 h in assay buffer, containing 3 nM [^3^H]PK11195 (specific activity 82.7 Ci/mmol; NET885, PerkinElmer). To determine non-specific binding (NSB), adjacent sections were incubated with 3 nM [^3^H]PK11195 in the presence of 10 μM unlabeled PK11195 (C0424; Sigma-Aldrich). Incubations were terminated by three 1-min washes into ice-cold 50 mM Tris–HCl buffer (pH 7.4), followed by a rapid rinse in ice-cold deionized H_2_O (dH_2_O). The sections were rapidly dried and laid down to Carestream^®^ Kodak^®^ BioMax MR film for 4 weeks. To allow quantification, ^3^H microscales of known radioactive concentration were also exposed to film (American Radiolabeled Chemicals, Inc). The films were developed with KODAK substitute D-19 developer (TED PELLA, Inc), washed in dH_2_O, and fixed in Carestream^®^ autoradiography GBX fixer. Images were digitized using a white sample tray and the Coomassie Blue settings on a ChemiDoc^TM^ MP imaging system (BIO-RAD). Values of specific binding were derived after subtraction of non-specific from total binding images, using ImageJ software.

For SV2A, sections were incubated for 2 h in assay buffer, containing 1 nM [^3^H]UCB-J (specific activity 82.0 Ci/mmol; NT1099, NOVANDI Chemistry AB). NSB was determined in the presence of 500 μM Levetiracetam (TOCRIS). The films were developed after 5 weeks of exposure using a PROTEC OPTIMAX 2010 automatic film processor. All remaining procedures were as described for TSPO autoradiography.

### Statistical Analysis

Data were analyzed with GraphPad Prism (v. 8.2.0; GraphPad Software), using non-parametric statistics. Age, brain weight, 6E10- and AT8-positive area fractions, [^3^H]UCB-J and [^3^H]PK11195 binding levels, were compared between AD and non-AD subjects by unpaired, two-tailed Mann-Whitney *U* tests. Spearman’s correlation was used to examine the association between radioligand binding levels and 6E10- or AT8-positive area fractions in the gray matter of AD patients. In all cases, the significance level was set at 5%. Results are presented as the median and interquartile range of *n* = 7 subjects/group.

## Results

### Prevalence of Aβ and pTau Pathology

**FIGURE 1:**
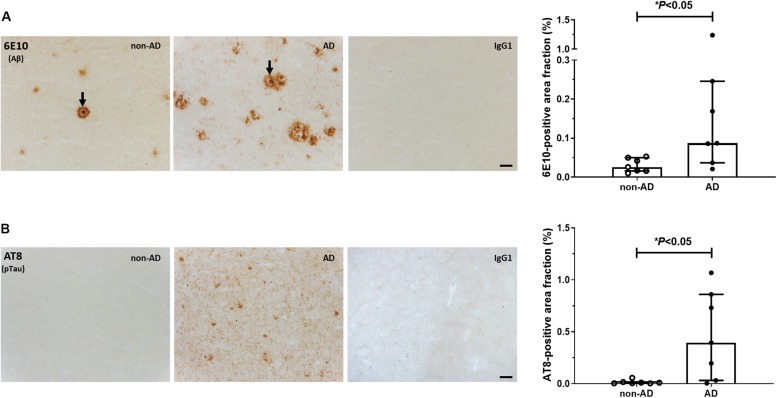
Immunohistochemical analysis of Aβ and pTau. Representative photomicrographs and quantification of 6E10-positive Aβ plaques **(A)** and AT8-positive pTau lesions **(B)** in the middle frontal gyrus of non-demented subjects and AD patients. Arrows in **(A)** point to dense-core plaques. No signal was observed in the IgG1 isotype controls. Levels of Aβ and pTau immunoreactivity were higher in AD vs. non-AD subjects (^∗^*P* < 0.05, Mann-Whitney *U* tests, two-tailed). Results are presented as the median and interquartile range of *n* = 7 subjects/group. Scale bars: 50 μm.

Representative photomicrographs of Aβ and pTau immunostainings are shown in Figure 1. Dense-core plaques were present in all AD cases and in 3 out of 7 non-demented individuals (No. 10, 12, 14; Figure 1A). Variable levels of diffuse, ill-contoured deposits were present in all subjects. There was no association between age at death and 6E10 immunoreactivity (Spearman *r* = −0.07, *P* = 0.82). The 6E10-positive area fraction was higher in AD patients compared to non-AD subjects (*U* = 7.0, *P* < 0.05). For pTau, AT8-immunoreactive tangles and threads were exclusively observed in material from AD patients (*U* = 6.5, *P* < 0.05; Figure 1B). There was no significant association between age at death and the percent area occupied by AT8 immunoreactivity (Spearman *r* = −0.57, *P* = 0.20).

### Increased [^3^H]PK11195 Binding Levels in AD

**FIGURE 2:**
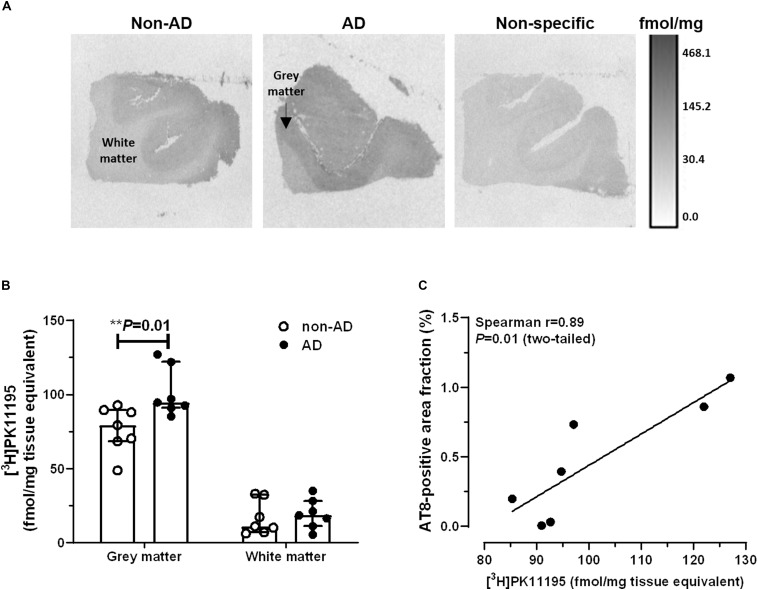
Autoradiography of [^3^H]PK11195 binding sites. **(A)** Representative autoradiograms of TSPO binding sites in the middle frontal gyrus of non-demented subjects and AD patients. The scale bar represents an interpretation of black and white image density, calibrated in fmol/mg of tissue equivalent. **(B)** Increased binding levels were observed in the gray matter of AD patients, compared to non-AD subjects (^∗∗^*P* = 0.01, Mann-Whitney *U* tests, two-tailed). **(C)** In AD patients, levels of [^3^H]PK11195 binding were positively correlated with pTau immunoreactivity. Results are presented as the median and interquartile range of *n* = 7 subjects/group.

Representative autoradiograms of [^3^H]PK11195 binding sites are shown in Figure 2A. Specific binding amounted to 63% of total binding levels and was primarily observed in the gray matter. There were increased [^3^H]PK11195 binding levels in the gray matter of AD patients compared to non-demented subjects (*U* = 5.0, *P* = 0.01; Figure 2B). No between-group differences were observed in the white matter (*U* = 19.0, *P* = 0.52). In the gray matter, [^3^H]PK11195 binding density was positively correlated with AT8 immunoreactivity (Spearman *r* = 0.89; *P* = 0.01; Figure 2C). There was no correlation between levels of [^3^H]PK11195 binding and the Aβ-immunoreactive area fraction (Spearman *r* = −0.28; *P* = 0.33).

### Unaltered [^3^H]UCB-J Binding Levels in AD

**FIGURE 3:**
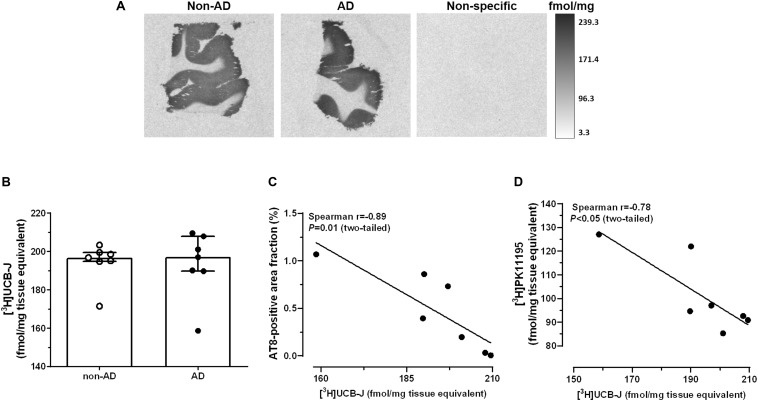
Autoradiography of [^3^H]UCB-J binding sites. **(A)** Representative autoradiograms of SV2A binding sites in the middle frontal gyrus of non-demented subjects and AD patients. **(B)** There were no differences in [^3^H]UCB-J binding levels between AD and non-AD subjects (*P* > 0.05, Mann-Whitney *U* tests, two-tailed). **(C,D)** In AD patients, [^3^H]UCB-J binding density was negatively correlated with AT8 immunoreactivity **(C)** and [^3^H]PK11195 binding levels **(D)**. Results are presented as the median and interquartile range of *n* = 7 subjects/group.

Representative autoradiograms of [^3^H]UCB-J binding sites are shown in Figure 3A. Specific binding amounted to 81% of total binding levels and was exclusively observed in the gray matter. There were no differences in [^3^H]UCB-J binding levels between AD and non-demented subjects (*U* = 23.0, *P* = 0.87; Figure 3B). In AD patients, [^3^H]UCB-J binding density was negatively correlated with both AT8 immunoreactivity (Spearman *r* = −0.89; *P* = 0.01; Figure 3C) and [^3^H]PK11195 binding levels (Spearman *r* = −0.78; *P* < 0.05; Figure 3D). No correlation was observed between [^3^H]UCB-J binding and the Aβ-immunoreactive area fraction (Spearman *r* = −0.00; *P* = 0.99).

## Discussion

We have compared levels of the presynaptic marker [^3^H]UCB-J and the inflammation marker [^3^H]PK11195 between autopsy-confirmed AD patients and non-demented subjects in the middle frontal gyrus, a region that is vulnerable to Aβ deposition and atrophy in cognitively intact individuals (Oh et al., 2011; Fjell et al., 2014). While binding levels of [^3^H]UCB-J correlated with pTau load and [^3^H]PK11195 in AD patients, there was no difference in SV2A density between groups. In addition to Aβ and pTau, increased levels of the inflammatory marker TSPO were observed in AD patients vs. non-AD subjects.

Biomarker studies highlight the early involvement of amyloid in the pathologic changes of AD. In longitudinal investigations (Sutphen et al., 2015), the concentration of soluble Aβ42 is decreased in the cerebrospinal fluid (CSF) of cognitively intact subjects, starting in the early middle-age (45–54 years). The reduction is associated with the aggregation and subsequent deposition of Aβ42 into cerebral plaques (Vlassenko et al., 2016). Positron emission tomography (PET) studies show that up to 35% of elderly individuals with normal scores in cognitive tests have fibrillar Aβ plaques in the brain (Villemagne et al., 2018). Thus, amyloid positivity is not only required for a definitive diagnosis of AD, but is also important for identifying asymptomatic individuals with neuropathologic evidence of AD (Jack et al., 2018). In our small cohort of non-demented cases, dense-core plaques were observed in 3 out of 7 subjects, a proportion that is within the range of amyloid positivity reported by PET studies. Diffuse Aβ deposits, however, were detected in all cases, irrespective of dementia state. Although the pathological (Abner et al., 2018) and practical (Ikonomovic et al., 2018) significance of diffuse Aβ is being investigated, these observations imply that the prevalence of amyloid positivity among people without dementia may be higher than what is currently being detected by imaging biomarkers. Similarly, tau imaging agents are unlikely to detect AT8-positive pretangle material, which is present in all individuals by the 5th decade of life, primarily in subcortical regions (Braak et al., 2011). Determining how these pervasive neuropathologic changes culminate to dementia in the AD continuum will require longitudinal studies and the earliest detection of disease-relevant biomarkers.

Levels of TSPO are low in the neuropil under physiological conditions, but increase in response to acute or chronic injury, rendering TSPO a key biomarker of inflammatory processes in the brain. Although not a universal finding (Xu et al., 2019), most autoradiography studies indicate that the binding of [^3^H]PK11195 is elevated in the postmortem frontal cortex of AD patients compared to non-AD subjects (Diorio et al., 1991; Venneti et al., 2009). Increased uptake of [^11^C]PK11195 in the AD brain has been also reported by several imaging studies (reviewed in Edison et al., 2018). The increased TSPO signal may reflect both pro- and anti-inflammatory processes, depending on age (Schuitemaker et al., 2012), AD stage (Fan et al., 2017) and the dynamic roles that TSPO-expressing glia play in the course of disease (Guilarte, 2019). In the present study, [^3^H]PK11195 binding levels were associated with increased pTau load and reduced SV2A density in the AD group, indicating that the elevated TSPO signal is likely representative of a pro-inflammatory environment. Our observations are in line with studies showing that microgliosis and astrocytosis correlate positively with the burden of neurofibrillary tangles in the AD brain (Serrano-Pozo et al., 2011). They are further consistent with longitudinal PET studies, showing that TSPO levels correlate positively with tau aggregation (Dani et al., 2018), and negatively with synaptic function in AD (Fan et al., 2015). These findings imply that reducing inflammation could play a beneficial role in attenuating tau pathology and synaptic dysfunction in AD. It should be mentioned that a positive correlation between TSPO and pTau immunoreactivity was not observed in the postmortem temporal cortex of AD patients (Gui et al., 2019), suggesting that the interplay between inflammation and tau pathology may occur in a region-specific manner.

In agreement with results from SV2A imaging studies in AD patients (Chen et al., 2018) and models of AD (Toyonaga et al., 2019), we observed no differences in the neocortical binding levels of [^3^H]UCB-J between AD and non-AD subjects. As AD is a neurodegenerative disorder, several mechanisms have been put forward to explain the apparent preservation of neocortical presynaptic elements in [^11^C]UCB-J PET studies. These include compensatory mechanisms, which can maintain the numbers of synaptic vesicles in the frontal cortex of AD patients (Scheff and Price, 2006), as well as mechanisms that may obscure the extent of SV2A loss in the plaque-rich AD neocortex (Snow et al., 1996). In addition, while SV2A is equally expressed by excitatory and inhibitory synapses (Gronborg et al., 2010), evidence suggests that there is preferential loss of glutamatergic rather than GABAergic nerve terminals in AD (Kirvell et al., 2006; Govindpani et al., 2017). This asymmetric loss may reduce the ability of [^11^C]UCB-J to detect decreases in SV2A density. Of note, SV2A-targeting drugs have been shown to preferentially disrupt GABAergic neurotransmission in epilepsy studies (Ohno and Tokudome, 2017). Additional explanations for the unchanged levels of SV2A density in this study include the presence of SV2A protein in mitochondria (Stockburger et al., 2016), which may mask reductions in SV2A levels in synaptic vesicles, and the fact that not all presynaptic proteins are equally reduced in AD (Poirel et al., 2018). For example, “general” markers of the presynaptic compartment, such as synaptophysin, are relatively spared compared to neurotransmitter-specific markers, even at the late AD stages. Moreover, synapses in Brodmann area 46 are known to be particularly susceptible to the effects of aging. Electron microscopy studies indicate that aging reduces the density of synapses in the primate prefrontal cortex by at least 30% (Morrison and Baxter, 2012). This extensive physiological reduction may explain why meta-analysis reveals limited decrease of synapse numbers in the postmortem frontal cortex of AD patients compared to age-matched, non-AD subjects (de Wilde et al., 2016). Despite comparable [^3^H]UCB-J binding levels between AD and non-AD cases in our study, the observation that SV2A density was inversely correlated with increases in tau phosphorylation and neuroinflammation, indicates that SV2A levels are regulated by AD-associated processes.

## Conclusion

In conclusion, we have examined markers of neuroinflammation and synapses in the middle frontal gyrus of autopsy-confirmed AD patients and non-demented subjects. Our small exploratory study provides evidence of tight associations between inflammation levels, tau pathology, and SV2A density in AD. Studies with larger sample sizes, including more brain regions, are warranted.

## Data Availability Statement

The raw data supporting the conclusions of this article will be made available by the authors, without undue reservation, to any qualified researcher.

## Ethics Statement

The study was carried out in accordance with the recommendations of the Danish Biomedical Research Ethical Committee for the Region of Southern Denmark (Project Id. S-20160036) and the Nova Scotia Health Authority Research Ethics Board in Halifax, NS, Canada. Written, informed consent forms were obtained for all subjects, in accordance with the Declaration of Helsinki. Samples were transported to the University of Southern Denmark from the Maritime Brain Tissue Bank, Department of Medical Neuroscience, Faculty of Medicine, Dalhousie University, Halifax, Canada.

## Author Contributions

AM wrote the manuscript and performed the SV2A autoradiography and data analysis. CT performed the immunohistochemistry experiments. SB performed the tissue sectioning and TSPO autoradiography. AL and SD provided the reagents and tissue. BF supervised the project. All authors made substantial contributions to study design, participated in drafting and critically reviewing the manuscript, and approved its final version.

## Conflict of Interest

The authors declare that the research was conducted in the absence of any commercial or financial relationships that could be construed as a potential conflict of interest.
